# Football

**Published:** 1897-12

**Authors:** 


### EDITORIAL.

---

The office of the Business Manager of THE JOURNAL is 311 and 312 Fitten Building.

The editors are not responsible for views expressed by contributors.

Articles for publication should reach this office not later than the 15th inst.

Address all communications, and make all remittances payable to THE ATLANTA MEDICAL AND SURGICAL JOURNAL, Atlanta, Ga.

Reprints of articles will be furnished, when desired, at a small cost. Requests for the same should always be made *on the manuscript*.

---

### FOOTBALL.

A longer list of casualties will be charged up to this popular knock-down and drag-out game for 1897 than for any preceding year.

To say nothing of the large number of such insignificant injuries as sprains, contusions and fractures, there have been several deaths on the football field within the last three months, one of which occurred in this city last month. These accidents, more or less severe, occurring with such alarming frequency, have dampened the ardor of former lovers of the brutal sport and have stimulated the legislatures in some States to initiate prohibitory laws against it. The lower house of the Georgia legislature has passed such an act unanimously and without debate, and we think public sentiment in this State will indorse the action.

The *North Carolina Medical Journal* (November 20th) contains an editorial on this subject, which is to the point and full of hard sense:

"We earnestly advocate the encouragement of athletics among young men and also young women, and especially is this necessary for students. Every college should be provided with a well equipped gymnasium, and field sports should also be encouraged. An hour or two spent daily in the gymnasium would be of immense benefit, especially to hard students, not only by improving their physical development, but in putting their minds in a more healthful and receptive condition. Moderate, regular exercise is helpful; spasmodic, violent exertion is harmful and dangerous. The 'rush' in a game of modern football requires the players to exert themselves to their utmost; they must strain every nerve to penetrate the opposing line, the members of which are taxed to their utmost capacity to resist the attack. So dangerous are these scrimmages that the players now try to protect themselves from accidental and *intentional* blows by armor which falls only a little short of the coat of mail of the middle ages.

"The game is not one of skill or science, for it is pure, unadulterated brute force that wins the battle. The very men who need especially the physical training through which the players pass are the ones which are studiously avoided in forming the teams. Only those of fine physique are desired, and they are not likely to apply themselves so diligently to their studies that their bodily health will be endangered. It is not our place to discuss the question as to the wisdom of permitting young men who are sent to college at considerable expense by their parents, and sometimes by the taxpayers, absenting themselves from their studies to travel for weeks from town to town and State to State to play football. Our object is to enter an absolute protest against the game as a sport which has no redeeming features. It is brutal, for there is seldom a game in which one or more players do not have to be carried from the field; it is dangerous to life and limb, as the yearly records of dozens of deaths and scores of serious injuries will demonstrate; it is unmanly, as any one must admit who has seen the heap of writhing human forms struggling in the dust and dirt after the manner of fighting beasts."

